# Supplementary material for: Deep learning for twelve hour precipitation forecasts
Source: Nat Commun. 2022 Sep 1;13:5145. doi: 10.1038/s41467-022-32483-x (PMC9436943; doi:10.1038/s41467-022-32483-x)
Supplement: Supplementary file 1 — Supplementary Information [file 41467_2022_32483_MOESM1_ESM.pdf]

# Appendix to
# *Deep learning for Twelve Hour Precipitation Forecasts*

Lasse Espeholt[1*], Shreya Agrawal[1], Casper Sønderby[1], Manoj Kumar[1], Jonathan Heek[1], Carla Bromberg[1], Rob Carver[1], Marcin Andrychowicz[1], Cenk Gazen[1], Jason Hickey[1], Aaron Bell[1], and Nal Kalchbrenner[1*]

[1]*Google Research, Google Inc*
[*]*equal contribution*

## A Supplement: Related Work

Operational weather forecasts based on NWP rely on decades-long research using laws of physics for atmospheric simulation. While the tremendous increase in observational data, scientific and computing advancements has resulted in an increase in the forecast skill by about one day every decade [4], the recent success of deep learning in scientific domains has spurred an interest in its application to weather forecasting [9, 27]. For the domain of nowcasting, Prudden et al. [19] provide a detailed review of radar-based nowcasting techniques and several ML approaches used in the past. Shi et al. [33] applied a recurrent neural network based approach using convolutional LSTMs [13]. Agrawal et al. [2] use a U-Net model and turn the forecasting problem into an image-to-image translation problem. Trebing et al. [31] apply attention modules and show a reduction in model parameter size while maintaining performance. Ravuri et al. [21] use a generative model that is radar-based only. However, all of these techniques have been shown to be skillful only from 0 to at most 3 hours of lead time. The MetNet model [28] shows initial results for for skilful nowcasting of precipitation up to 7-8 hours over a HRRR baseline. On the longer forecast sides, deep learning has also been applied for seasonal forecasts of weather events including extreme ones [11, 35].

As we move from nowcasting to short or medium range forecasting the biggest challenge is the lack of high resolution observational data that is typically obtained through data assimilation. There have also been efforts to postprocess NWP predictions using neural networks by incorporating them into the set of input features [20]; this is similar to our MetNet-2 Postprocess variant.

## B Supplement: Dataset

The training data consists of 1,230,585 patches of size 2048 km $\times$ 2048 km at the input and targets of size 512 km $\times$ 512 km including all 360 (2 to 720 minutes) time slices. The training area covers a region of 7000 $\times$ 2500 kilometers. We sample target patches from the input context region minus an all around border of $\approx$512 km at fixed locations spaced .5 degrees from each other. The input context is padded for all regions outside of the 7000 $\times$ 2500 CONUS. The validation data used for developing the models consists of 11,991 patches and the two test datasets consist of 39,864 patches each. The test dataset A has only cumulative precipitation targets, whereas the test dataset B has both instantaneous and cumulative precipitation targets. The training, validation and test data are drawn from non-overlapping ranges of hours, with “black out” periods of 12 hours in between, over a period of observations of 3 years from July 2017 to August 2020. This ensures that the model does not learn any spurious training and evaluation correlations within any single day. HREF and HRRR only generate forecasts starting at full hours; the available HREF forecasts start times are a subset of those of HRRR.

| Input Sets | #Features | Resolution (km) | Frequency (min) |
|---|---|---|---|
| Radar (MRMS Instantaneous) | 1 | 1 | $\approx$2 |
| Gauge-corrected Radar (MRMS Cumulative) | 1 | 1 | 60 |
| GOES Optical Images | 16 | 1 | $\approx$15 |
| Assimilation | 612 | 3 | 60 |
| Geospatial coordinates | 3 + 3 | 1 | 1 |
| NWP Forecast for MetNet-2 Postprocess/Hybrid | 2 | 3 | 60 |

Supplementary Table 1: For each set of inputs to MetNet-2, we list the respective number of features, the native resolution of the data and the temporal frequency by which each set of features is generated.

## B.1 Data Types

MetNet-2's radar data comes from the Multi-Radar Multi-Sensor (MRMS)[17] data that uses the reflectivity of ground radars and processes it to estimate precipitation. The data has a spatial resolution of approximately 1 km $\times$ 1 km and a temporal resolution as low as 2 minutes. MRMS provides both of the precipitation measures that we use: the instantaneous rate of precipitation, that we refer to here as MRMS Instantaneous, and the hourly cumulative precipitation, that we refer to as MRMS Cumulative. The MRMS Cumulative estimates use rain gauges at weather stations to further corroborate the radar measurements. For ensuring as precise evaluation as possible, we evaluate only regions where radar measurements are of high quality (Supplementary Figure 1). For training we use a larger mask corresponding to the full range of the radars.

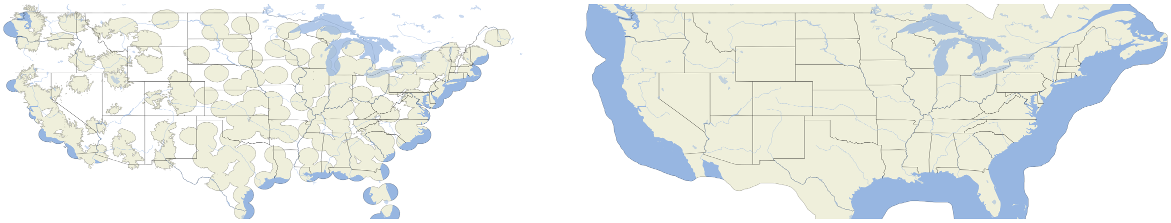

Supplementary Figure 1: Continental United States masked using the "better" range of NOAA NEXRAD on the left for evaluation and the training mask "roi" on the right.

The NWP model HRRR also provides atmospheric observations at forecast time $t_0$ that is a result of an hourly updated data assimilation system [5]. We use 612 of these variables that capture atmospheric and oceanic variables such as pressure at various altitudes in the atmosphere, humidity, wind velocity and direction, temperature and current precipitation levels [14]. The data generally originates from sparse empirical measurements but the result of the data assimilation process is a dense grid with a spatial resolution of 3 km $\times$ 3 km.

Additional input types include images from the GOES [10] weather satellites. These come in 16 optical bands, have a resolution of approximately 1 km $\times$ 1 km and a temporal frequency of $\approx$15 minutes. Furthermore, simple geographical features for longitude, latitude, elevation [28] and temporal features that encode the forecast time $t_0$ are additional sources of inputs for the MetNet-2 models.

Finally, the precipitation forecast from the NWP (HRRR) model is also used as input in the MetNet-2 Postprocess and Hybrid variants. These are two features that include an instantaneous rate forecast and an hourly cumulative forecast that correspond to the same measures, respectively, as MRMS Instantaneous and MRMS Cumulative. Supplementary Table 1 summarizes the sets of input features and their main characteristics.

## B.2 Distribution of Precipitation Events

The target data on which we train and evaluate MetNet-2 and its variants comes from the MRMS Instantaneous and MRMS Cumulative radar measures. The distribution of the levels of precipitation is summarized

| | Precipitation Bucket (mm/hr) | | | | | | |
|---|---|---|---|---|---|---|---|
| **Measure Type** | 0 | .2 | 1 | 2 | 4 | 8 | ≥20 |
| MRMS Instantaneous | 93.83 % | 3.23 % | 1.43 % | .92 % | .41 % | .13 % | .06 % |
| MRMS Cumulative | 92.74 % | 4.24 % | 1.49 % | .92 % | .42 % | .17 % | .03 % |

Supplementary Table 2: Distribution of precipitation levels in test data for the two precipitation measures.

in Supplementary Table 2. The specific numbers are from the test data within the evaluation mask (Supplementary Figure 1), but the training data distribution is similar. Around ≈93 % of precipitation events corresponds to no precipitation of 0 mm/hr. Higher amounts of precipitation events get increasingly more rare. This has consequences for learning too, as the MetNet-2 needs to learn good estimates for higher levels of precipitation from a relative paucity of data. During training, we resample the data to contain higher amounts of precipitation. Each training patch is ranked according to the average precipitation of all targets in the patch. Prioritized resampling is inspired by Prioritized Experience Replay [25]: a priority exponent of 0 induces a uniform sampling of all the patches without prioritization. We find experimentally that an exponent of 2 works well and leads to faster convergence for the MetNet-2 models. With long enough training though a uniform exponent reaches the same performance.

# C Supplement: Evaluation Metrics

We evaluate the quality of the precipitation forecasts using three different metrics, the Critical Skill Index (CSI) [22], the Brier score [8] and the Continuous Ranked Probability Score (CRPS) [12].

## C.1 Critical Success Index

The CSI score is a binary categorical score similar to the F1 score that goes beyond plain accuracy to take into account more aspects of the confusion matrix. CSI is defined as follows:

$$CSI = TP/(TP + FN + FP) \tag{2}$$

where TP are true positives, FN are false negatives and FP are false positives. Like the F1 score, the CSI score is not directly applicable to the probability distributions that MetNet-2 produces. To make a categorical decision, for a binary category corresponding to an amount of precipitation greater or equal to a given rate $r$, we calculate on held-out data a probability threshold between 0 and 1. If the total predicted probability mass for rates $\geq r$ exceeds the threshold, then we take it to be a positive prediction for this rate category. This is the same procedure as used for the F1 score in Sønderby et al. [28]. We calculate the CSI scores of MetNet-2 on multiple rates ranging from small amounts of precipitation (0.2 mm/hr) to high amounts of precipitation (20 mm/hr).

## C.2 Brier Score

The Brier score by contrast is not categorical and measures the magnitude of the error between the ground truth rate and the probability that a model predicts for that rate:

$$BS_r = \frac{1}{N}\sum_{n}(P(r) - \mathbb{1}(y \geq r))^2 \tag{3}$$

where $y$ is the ground truth rate at the respective time and location and $\mathbb{1}$ is the indicator function. We calculate the Brier score from the raw probabilities produced from the output of MetNet-2 and do not calibrate the learnt distribution after training, which can affect the absolute values of the Brier score. For HRRR's deterministic forecast, we can also compute the Brier score by assigning probability 1 to the predicted rate value and 0 everywhere else.

| Architecture | Value |
|---|---|
| LSTM Channels | 128 |
| Encoder Blocks | 18 |
| Encoder Channels | 384 |
| Upsampler Blocks | 2 |
| Upsampler Channels | 512 |
| Lead Time Network - Layers | 2 |
| Lead Time Network - Features | 2048 |

| Training Hyperparamters | Value |
|---|---|
| Weight Decay | 0.1 |
| Polyak Decay | 0.9999 |
| Optimizer | Adam |
| Beta | 0.9 |
| Batch size | 16 |
| Learning rate | 2e-5 |
| Training steps | 500K |

Supplementary Table 3: Hyperparameters for MetNet-2 governing the architecture and the training procedure.

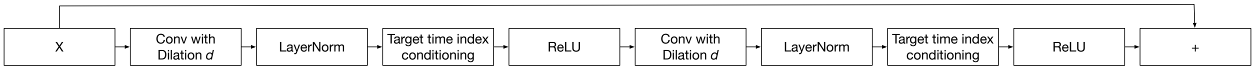


Supplementary Figure 2: Residual block composition in the MetNet-2 architecture.

### C.3 Continuous Ranked Probability Score

CRPS in essence is the mean squared error between the cumulative density function of the prediction and that of the ground truth. It can be defined as the Brier Score integrated over all rates, and therefore:

$$CRPS = \sum_{r=0.2}^{102.4} BS_r \times 0.2 \tag{4}$$

where we sum over all 512 discrete buckets of rate in increments of 0.2 mm/hr. Probabilistic metrics such as the Brier Score and CRPS are most appropriate for our evaluation as MetNet-2 and HREF are probabilistic models. The deterministic metric CSI provides additional insight into the models' ability to make categorical forecasts.

### C.4 Skill Scores

We compute skill scores for both Brier Score and CRPS. For both, we obtain skill scores with respect to HRRR's performance, as follows:

Brier Skill Score: $$BSS = 1 - \frac{BS}{BS_{NWP}}$$

Continuous Ranked Probability Skill Score: $$CRPSS = 1 - \frac{CRPS}{CRPS_{NWP}}$$

## D Supplement: Architecture and Training Details

We provide details about the hyper-parameters governing the architecture and the training procedure (see Supplementary Table 3 and Supplementary Algorithm 1). The MetNet-2's are trained in parallel on 64 TPU chips (128 TPU cores). It takes the models approximately 48 hours to train the convergence. We use weight decay to regularize the training and the models tend to converge faster on later lead times than on earlier lead times. Due to this imbalance, we perform per-lead-time-hour checkpointing, whereby we maintain the best checkpoints according to validation set performance for each hour of the 12 hours of lead time.

| Observations | Time Slices |
|---|---|
| Radar MRMS | 13 |
| Satellite GOES | 3 |
| Assimilation | 3 |
| Geospatial Coordinates | 1 |
| NWP Forecast | 1 |

Supplementary Table 4: Number of time slices used for each observation set.

## D.1 Rich Conditioning with Lead Time

The rich form of conditioning that MetNet-2 uses works as follows. After every convolutional layer in MetNet-2's encoding and upsampling residual blocks, two dense projections map the continuous representation of the lead time index into a bias and a scale vector. The bias and scale vector are different for each residual block. We add the bias to the output of every convolution at every position of the tensor and then multiply the result at each position with the scale vector. This ensures that the output of each convolutional layer now depends directly and very strongly on the lead time.

## D.2 Lead Time Conditioning for Hourly Cumulative Precipitation

For instantaneous precipitation, MetNet-2 gets a single lead time index to indicate the lead time minutes. When predicting hourly cumulative precipitation, the encoding is not a single one-hot embedding of just one index, but the 30 one-hot embeddings that correspond to all the 2 minute intervals in the hour preceding the lead time. This lets MetNet-2 share the same time indices for both targets and simultaneously training on the two targets while still being able to distinguish between the two targets. Per step, the combined model is on par with the individually trained models, signaling transfer learning between the targets.

**Algorithm 1** MetNet-2 Architecture

**procedure** FORWARDPASS$(x, t)$ //Sequence of 11 input frames of size 2048 by 2048 and lead time index
    $y \leftarrow SpatialMeanPooling(x, (4, 4))$ //Reduces resolution of the frames with new size 512 by 512
    $t \leftarrow MLPEmbedding(t)$ //Multi-layer embedding of lead time index
    $t \leftarrow Repeat(t, (1, 512, 512))$ //Upscale to spatial size of y
    $t\_time \leftarrow Repeat(t, (11, 1, 1))$ //Upscale to time dimension of y
    $y \leftarrow Concatenate([y, t\_time], axis = -1)$ //Concatenate context and lead time along features
    $c \leftarrow ConvLSTM(y, 256, (3, 3))$ //Temporal encoding of the frames
    **for** each step $l$ from 0 to 7 **do** //Spatial encoding
        $c \leftarrow ConditionedDilatedResidualBlock(c, t, 384, (3, 3), dilation = 2^l)$ //See Supplementary Figure 2
    **for** each step $l$ from 0 to 7 **do**
        $c \leftarrow ConditionedDilatedResidualBlock(c, t, 384, (3, 3), dilation = 2^l)$
    $s \leftarrow CenterCrop(c, (128, 128))$ //Crop to target region
    $s \leftarrow Repeat(s, (4, 4))$ //Repeat each value 4 by 4 times to obtain new size 512 by 512 (upsampling)
    **for** each step $l$ from 0 to 7 **do**
        $s \leftarrow ResidualBlock(s, 384, (3, 3))$ //Non-dilated residual blocks for processing after repeating
    $s \leftarrow Conv(s, 4096, (1, 1))$
    $s \leftarrow Relu(s)$ //Non-linearity
    $s \leftarrow Conv(s, 512, (1, 1))$
    $s \leftarrow SoftMax(s)$ //Softmax along channel dimension

# E Supplement: Forecasting Results

We perform an extensive evaluation of the models using the various metrics and for multiple levels of precipitation, both for the instantaneous measure and for the hourly cumulative. HREF, HRRR and MetNet-2 Postprocess are evaluated at every full hour, whereas MetNet-2 and MetNet-2 Hybrid at two minute intervals. One important thing to note is that we do not count the delay of the computation in our evaluations. Despite the fact that HRRR, HREF, MetNet-2 Postprocess and MetNet-2 Hybrid technically take approximately 60 minutes longer (or more in the case of HREF) than MetNet-2 to generate their forecasts due to the time used by the atmospheric simulations, we ignore this additional delay and do not consider it when comparing the performance based on lead time. HREF's and HRRR's prediction for $n$ minutes is compared with MetNet-2's prediction for $n$ minutes independently of the time it took to generate it and even if the generation time itself is longer than $n$ minutes. The same holds for the MetNet-2 variants that rely on the atmospheric simulation.

The results are summarized as follows. Figure 2 and Supplementary Figure 3 provide the CRPS and CSI that the models achieve for low and high rates for hourly cumulative precipitation. These use test dataset A and contain the comparison of HREF and MetNet-2 forecasts. To compare the MetNet-2 variants on both instantaneous and cumulative precipitation, Figure 4 in the main text gives numerical values for CSI for instantaneous rate $\geq$2 mm/hr, Figure 5a plots instantaneous rates of $\geq$2 mm/hr, $\geq$8 mm/hr and $\geq$20 mm/hr and Figure 5b gives the CRPS skill score at once for all levels of precipitation for the instantaneous precipitation. In this supplement, CSI, Brier Score and BSS for the instantaneous measure for additional levels of precipitation appear in Supplementary Figure 4, Supplementary Figure 6 and Supplementary Figure 5, respectively. For the hourly cumulative measure on the test dataset B, the CSI scores for additional levels of precipitation appear in Supplementary Figure 7 and the CRPS skill in Figure 5b. Note the difference in evaluation frequency for the various models.

## E.1 HRRR Reference

We include HRRR results throughout as reference. We evaluate HRRR with the natural categorical metric CSI and, for additional information, also with the CRPS metric by assigning probability 1 to HRRR's deterministic forecast. Two prominent features of HRRR's results is the relatively low performance for the first 2 hours of lead time and the positive flattening of the performance for the lead hours after that. HRRR's results on hourly rate forecasts paint a similar picture. The hourly cumulative precipitation estimates are calibrated with measurements from precipitation gauges making them less susceptible to biases directly linked to ground radar data. But this does not significantly affect the broad trend in HRRR's results, with a relatively low initial performance that flattens out over longer lead times into a relatively stronger performance.

## E.2 Ensemble NWP (HREF)

The probabilistic HREF model is a state-of-the-art NWP model for precipitation forecasts that ensembles 10 forecasts arising from 5 NWP models, including HRRR. We compute the best possible HREF thresholds for CSI by directly optimizing them on the test data itself (the thresholds on validation data turn out to be the same). HREF invariably improves over HRRR on both the CRPS and the CSI metrics, across all tested precipitation rates. The improvement is relatively equal across the whole lead time range.

## E.3 MetNet-2

Since it does not perform explicit atmospheric simulation, MetNet-2 needs to learn to approximate the underlying physics in order to make a good forecast. At longer lead times one requires a larger spatial context at the input patch and MetNet-2 must be able to capture that large context effectively. We can see the performance that MetNet-2 reaches in the tables of results. Compared with the probabilistic HREF, MetNet-2 outperforms HREF based on the probabilistic CRPS for the full 12 hour range. This uses hourly cumulative precipitation targets (on test dataset A). Using CSI and optimal thresholds for HREF, MetNet-2 is also superior to HREF up to nine or more hours for both low and high precipitation rates (Supplementary Figure 3). On instantaneous rate (on test dataset B), MetNet-2's performance is substantially better than

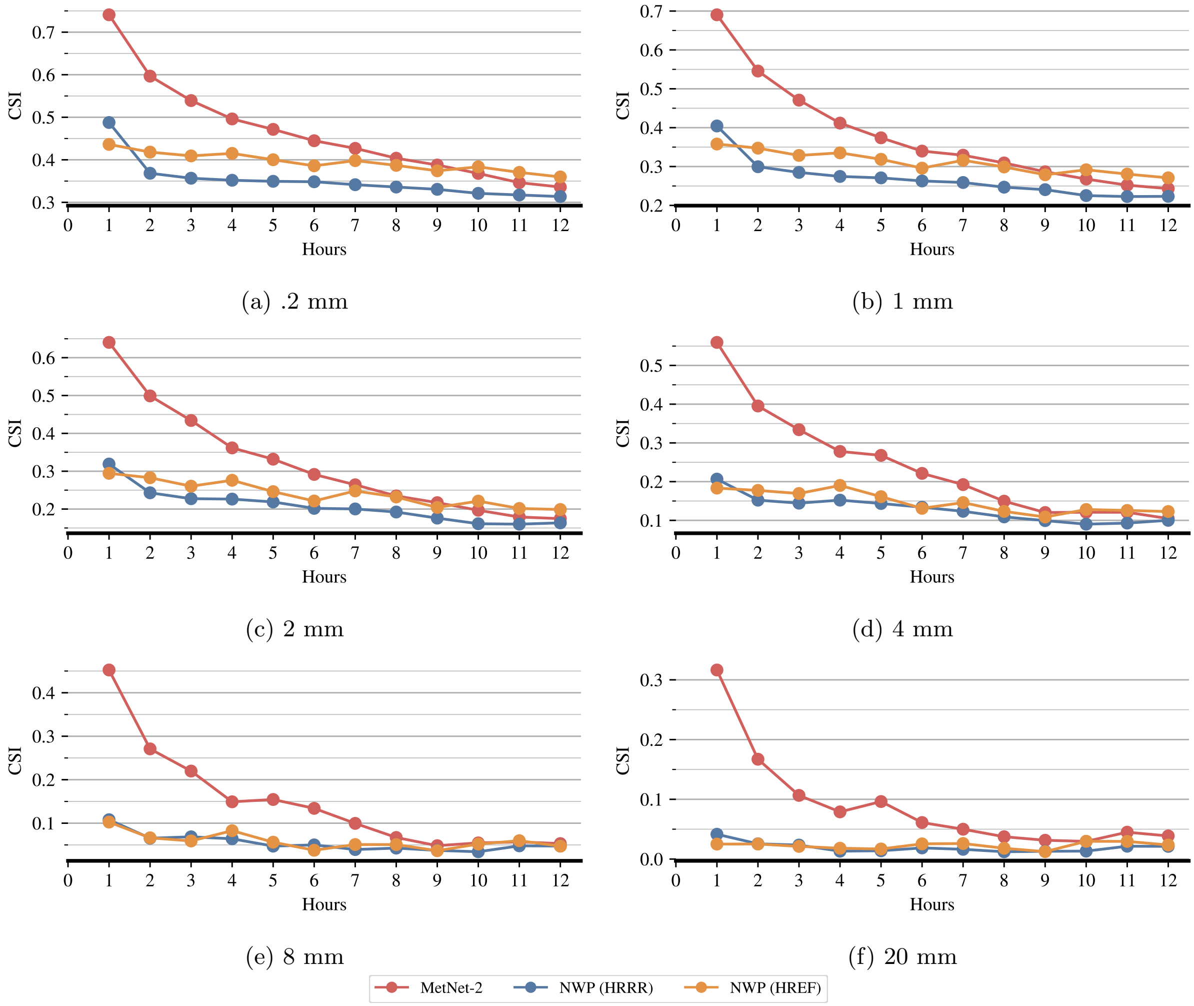


(a) .2 mm (b) 1 mm (c) 2 mm (d) 4 mm (e) 8 mm (f) 20 mm

Supplementary Figure 3: CSI performance of MetNet-2, the HREF ensemble and the reference HRRR for various low to high hourly cumulative precipitation rates of ≥.2 mm/hr, ≥1 mm/hr, ≥2 mm/hr, ≥4 mm/hr, ≥8 mm/hr and ≥20 mm/hr. Test dataset A.

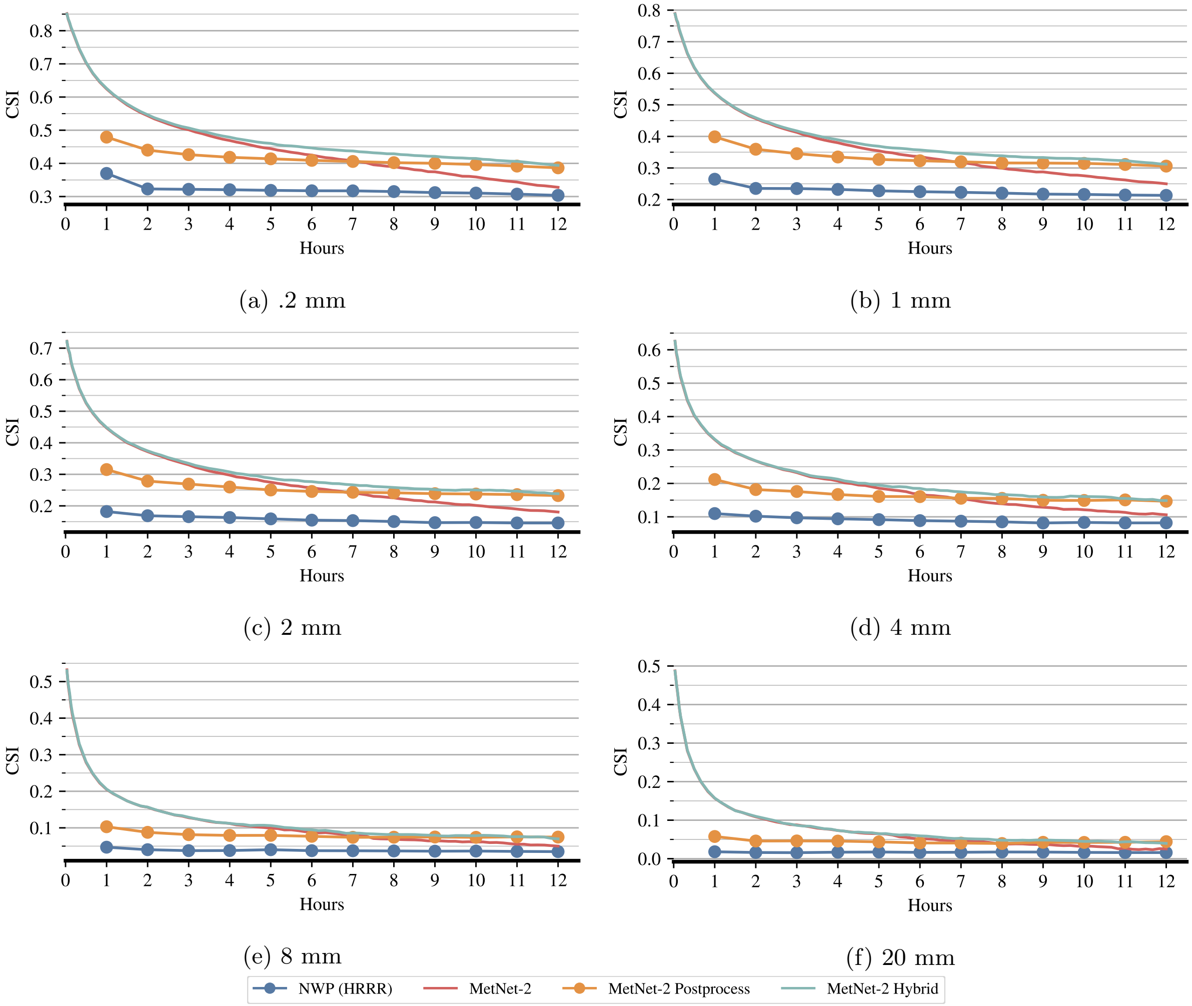


(a) .2 mm (b) 1 mm

(c) 2 mm (d) 4 mm

(e) 8 mm (f) 20 mm

Supplementary Figure 4: CSI performance of the MetNet-2 variants for various low to high instantaneous precipitation rates of ≥.2 mm/hr, ≥1 mm/hr, ≥2 mm/hr, ≥4 mm/hr, ≥8 mm/hr and ≥20 mm/hr, with default MetNet-2 and HRRR for reference. Test dataset B.

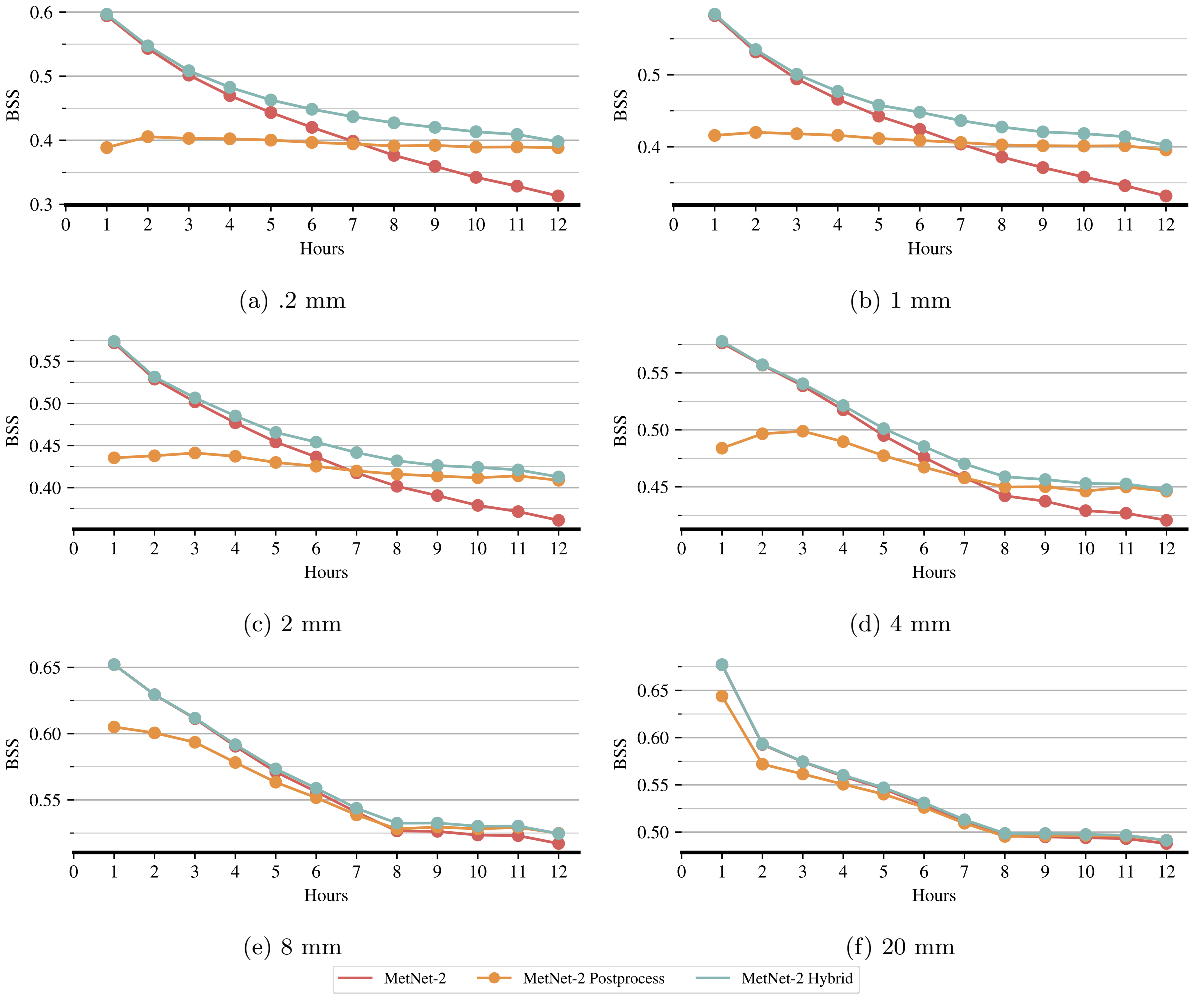


Supplementary Figure 5: Brier Skill Score relative to HRRR of the MetNet-2 variants (and MetNet-2 for reference) for various instantaneous precipitation rates of ≥.2 mm/hr, ≥1 mm/hr, ≥2 mm/hr, ≥4 mm/hr, ≥8 mm/hr and ≥20 mm/hr. HRRR's reference predictions are assigned probability 1. Test dataset B.

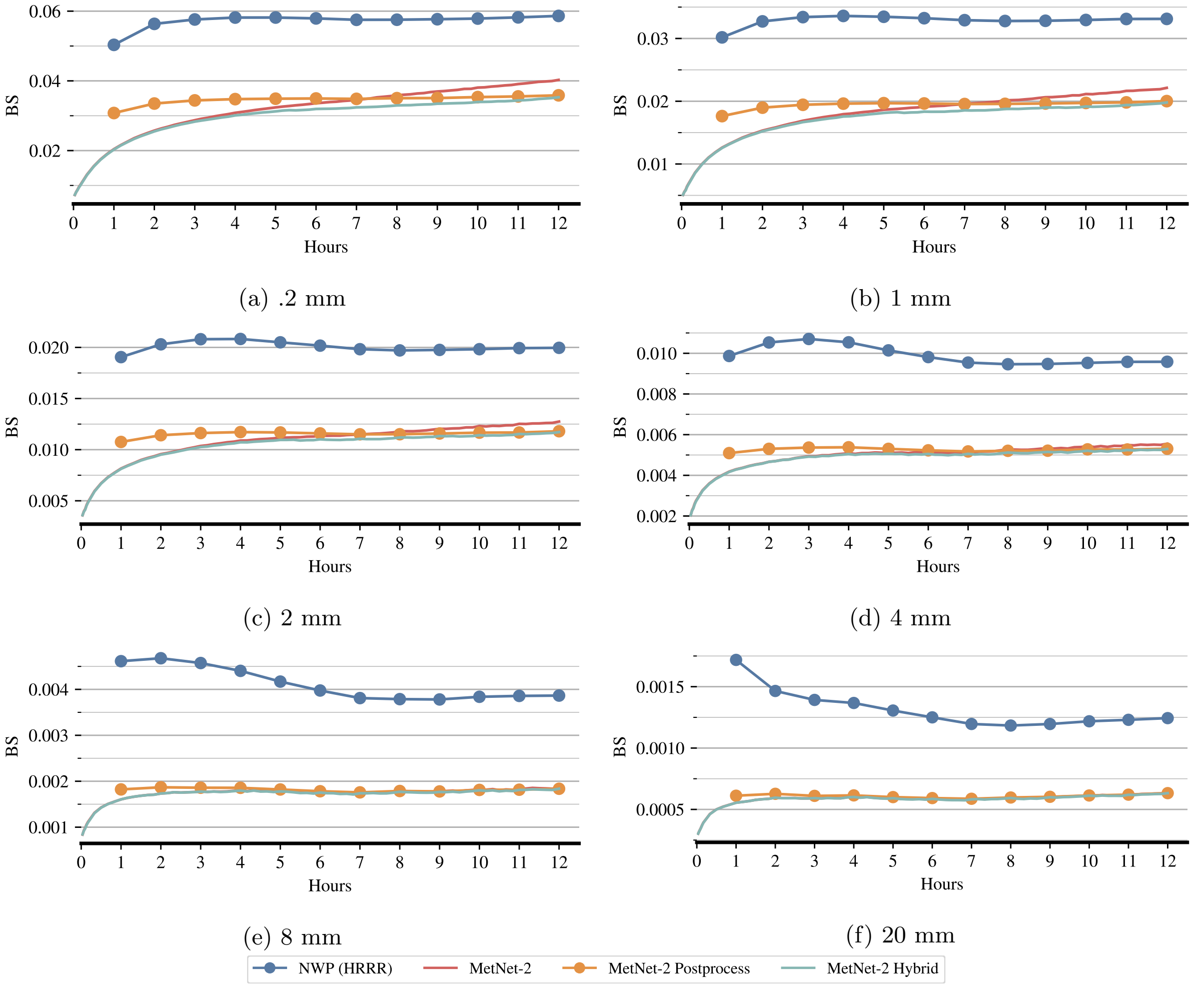


Supplementary Figure 6: Brier Score of MetNet-2 variants for various instantaneous precipitation rates of ≥.2 mm/hr, ≥1 mm/hr, ≥2 mm/hr, ≥4 mm/hr, ≥8 mm/hr and ≥20 mm/hr, with default MetNet-2 and HRRR as reference. HRRR's predictions are assigned probability 1. Test dataset B.

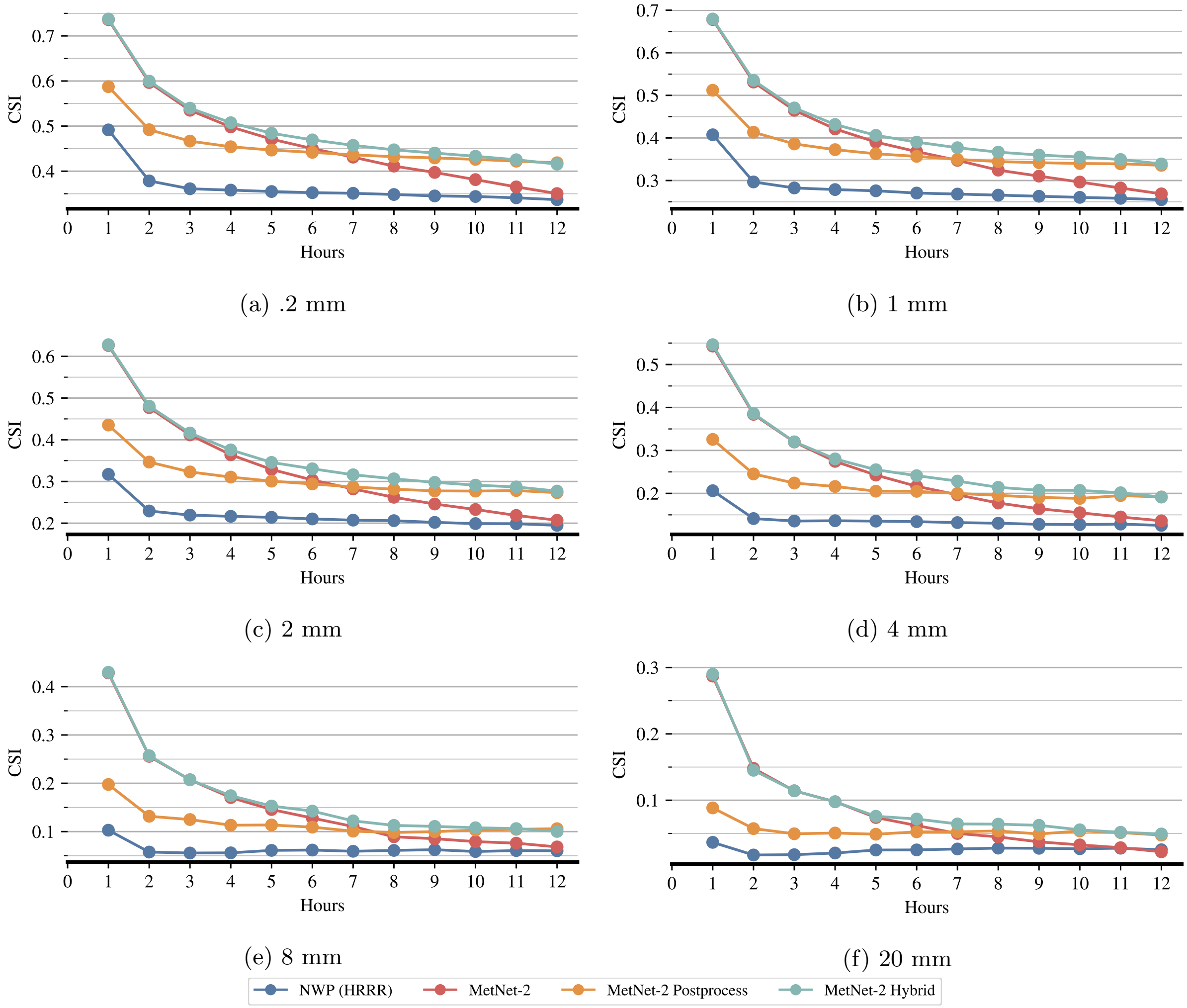


Supplementary Figure 7: CSI performance of MetNet-2 variants for hourly cumulative precipitation at rates of ≥.2 mm/hr, ≥1 mm/hr, ≥2 mm/hr, ≥4 mm/hr, ≥8 mm/hr and ≥20 mm/hr, with default MetNet-2 and HRRR as reference. Test dataset B.

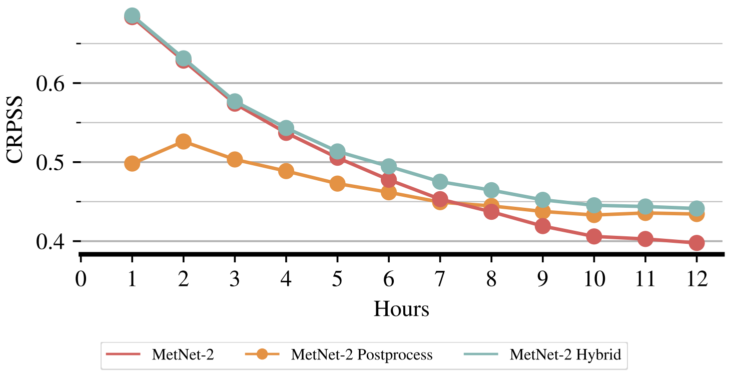


Supplementary Figure 8: Continuous Ranked Probability Score Skill for hourly cumulative precipitation for MetNet-2 variants, with default MetNet-2 for reference. The score tracks the relative improvement of MetNet-2 Postprocess and MetNet-2 Hybrid over HRRR. Test dataset B.

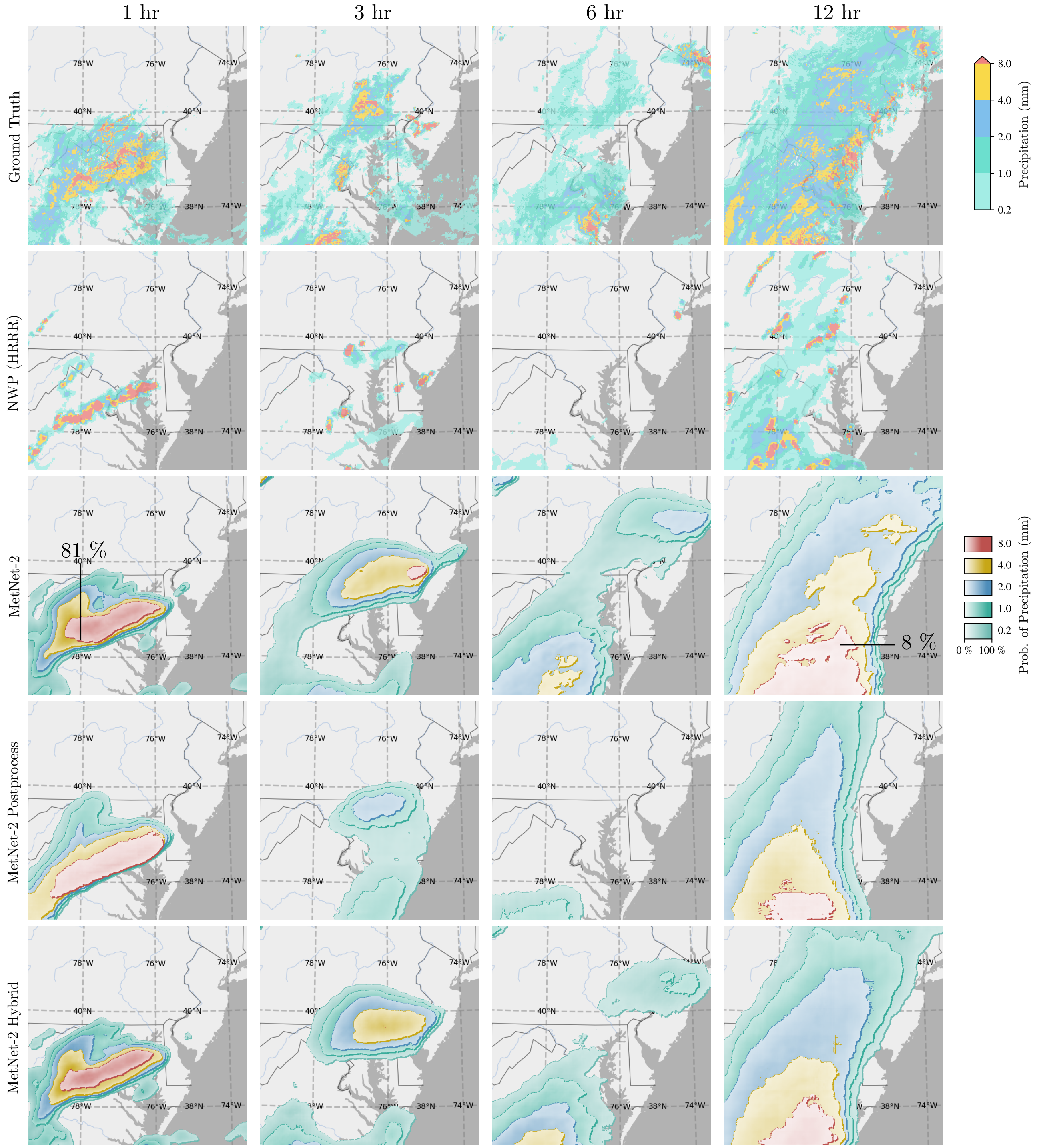


Supplementary Figure 9: Case study of Hurricane Isaias, a Category 1 hurricane. The forecast time is Mon Aug 03 2020 20:00 UTC on the East coast of the United States. The measure is the gauge-corrected hourly cumulative precipitation. We notice how MetNet-2 Hybrid combines information visible both in MetNet-2 and in MetNet-2 Postprocess.

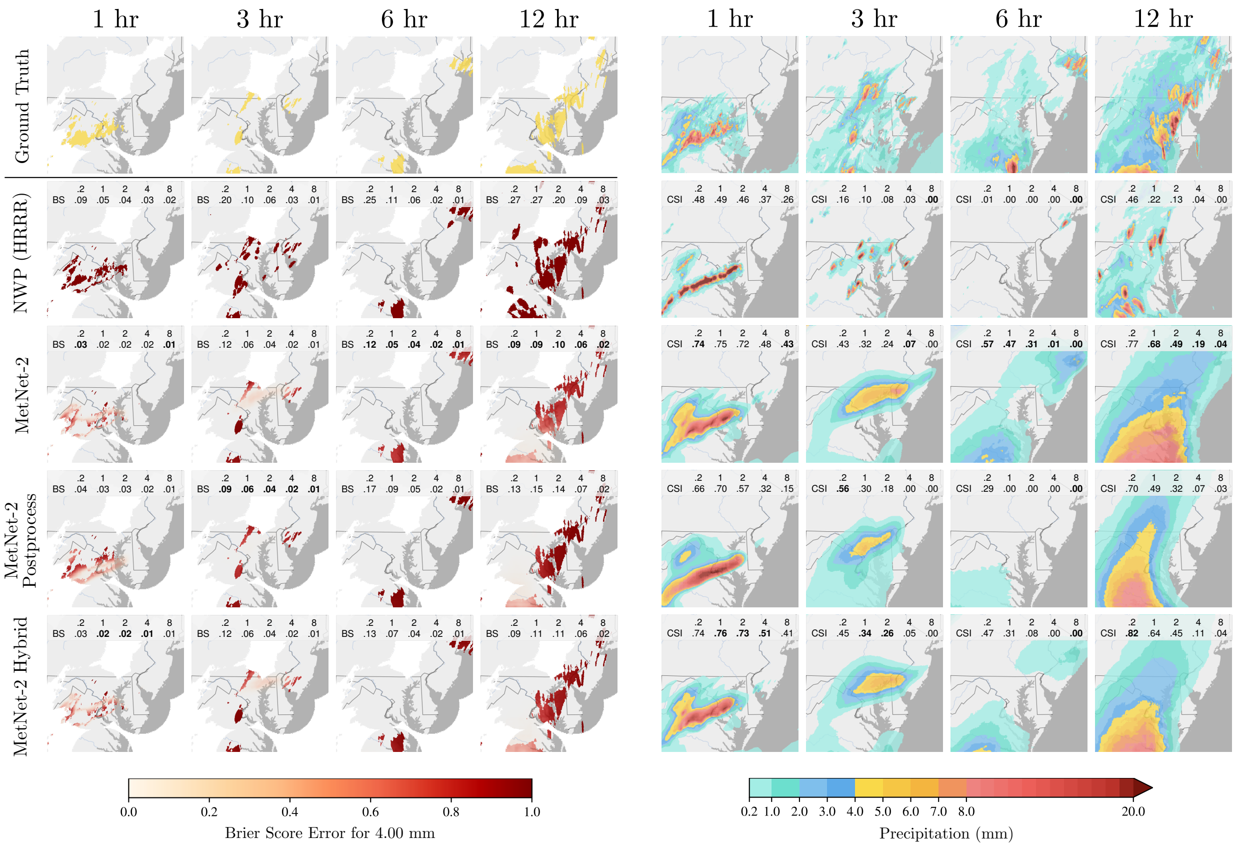


(a) Brier score maps that quantify the error of the probabilistic prediction. Lower error is better. HRRR's forecast is assigned probability 1 everywhere. Only the region of high quality radar signal is visualized (Supplementary Figure 1)

(b) CSI decision boundaries for hourly cumulative precipitation and respective scores.

Supplementary Figure 10: BS and CSI scores for the case study of Hurricane Isaiah.

HRRR's performance throughout the 12 hour range of lead time. The relative results of MetNet-2 remain similar across at low, medium and high instantaneous rates of precipitation up to 20 mm/hr, suggesting that MetNet-2 can learn to forecast also the much rarer rates for which data is much scarcer. Supplementary Figure 7 also shows the CSI scores for the hourly cumulative precipitation (on test dataset B) where MetNet-2's performance also exceeds that of HRRR over the 12 hour evaluation range.

### E.4 MetNet-2 Postprocess

MetNet-2 Postprocess learns to map the HRRR forecast to a probabilistic one. This yields both substantially better CRPS and CSI across the full range of the 12 hours. MetNet-2 Postprocess represents a baseline for what the architecture can achieve simply by postprocessing the HRRR forecast. For the first seven hours of lead time, MetNet-2 Postprocess is inferior than MetNet-2.

### E.5 MetNet-2 Hybrid

Our second core result is represented by the performance of MetNet-2 Hybrid. MetNet-2 Hybrid outperforms MetNet-2 Postprocess across the whole range of 12 hours, based on both CRPS and CSI, on both cumulative and instantaneous precipitation. The relative improvement is higher at the beginning, and becomes lower with lead time, but it is still substantial in relative terms even at 12 hours. This shows the ability of the MetNet-2 neural architecture to contribute additional useful information to a NWP forecast with a long lead time of twelve hours.

## F Supplement: Ablations

We provide a series of ablation studies to shed light on which aspects of MetNet-2's design drive its performance. These are run on test dataset B with instantaneous precipitation targets.

### F.1 Spatial Context

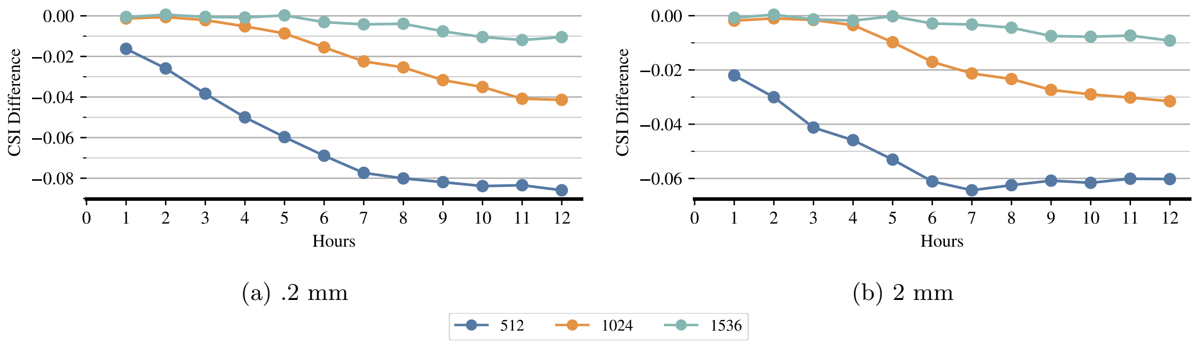


Supplementary Figure 11: Ablation experiments for the size of the input spatial context to MetNet-2, computed on instantaneous precipitation. Default size is 2048 by 2048 km. Smaller context size hurts performance especially at later hours.

A direct way of measuring the effect that context size has on forecast performance is by limiting directly the context size at the network's input. We do so for MetNet-2 by reducing the spatial context size from the default 2048 km $\times$ 2048 km centered around the target patch of 512 km $\times$ 512 km to 1536 km $\times$ 1536 km, 1024 km $\times$ 1024 km and 512 km $\times$ 512 km. Supplementary Figure 11 reports the results as a difference from the full context. We can see that reducing spatial input context incurs a marked performance drop that increases with the hours of lead time. The drop at 512 km $\times$ 512 km of spatial context already occurs at the earliest hours of lead time, suggesting that the model requires some context beyond the target patch even for short lead times. After 3 - 4 hours, MetNet-2 benefits from a spatial context that is larger than

1024 that corresponds to borders of 256 km around the target patch. After 5 - 6 hours, MetNet-2 benefits from an even larger spatial context of at least 1536 km that corresponds to 512 km around the target patch.

## F.2 Subsets of Assimilated Variables

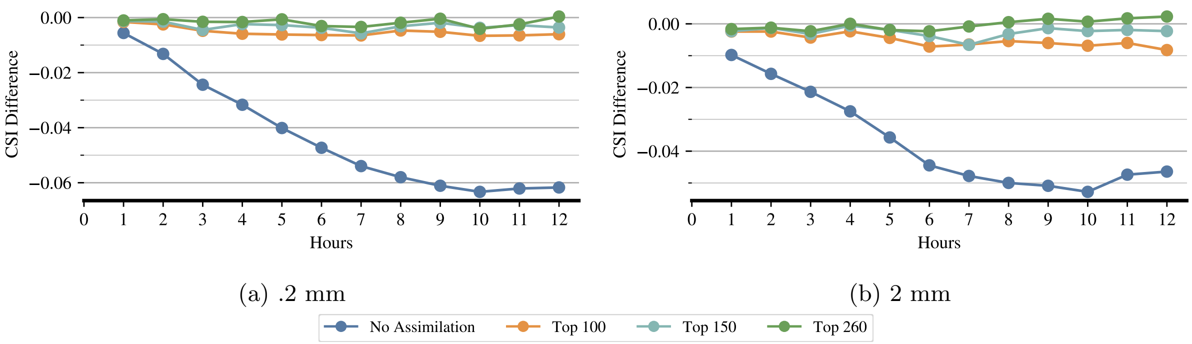


(a) .2 mm (b) 2 mm

Supplementary Figure 12: CSI score difference relative to MetNet-2 when using only subsets of the baseline 612 assimilation features: the top 260 features, the top 150 features, the top 100 features and none of the features (no assimilation). Results on instantaneous precipitation.

Besides ablating spatial context, we also run experiments by only using subsets of the Assimilation variables. The interpretability analysis (see Section G) allows us to approximately identify the importance of each variable and rank them accordingly. We then keep the top 260, top 150 or top 100 variables from the Assimilation state and evaluate their contributions. We also compare to the case of using none of the variables. Supplementary Figure 12 reports the results from the ablation experiments. Removing the less important Assimilation inputs degrades performance, again especially at later hours, suggesting the ability of MetNet-2 to gain useful signal even from less relevant variables. The case of not using any of the Assimilation variables incurs a significant drop in performance with respect to the default setting.

## F.3 Comparison with MetNet

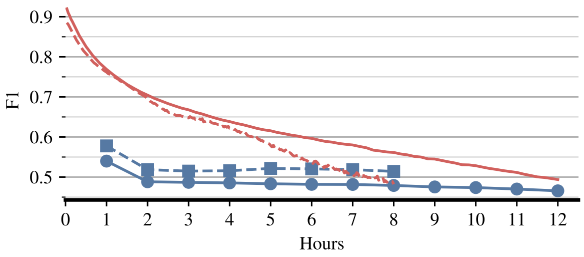


(a) .2 mm

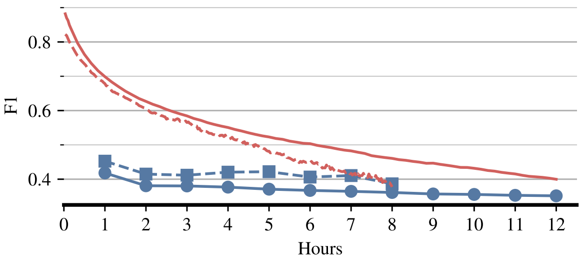


(b) 1 mm

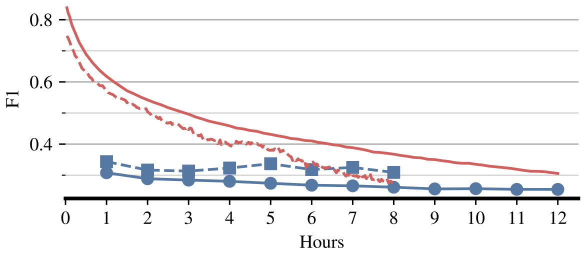


(c) 2 mm

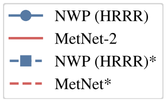


Supplementary Figure 13: Comparison using similar but distinct test sets of MetNet-2 and MetNet [28] on the originally used F1 metric, that is very similar to CSI. MetNet-2 outperforms MetNet substantially despite the fact that MetNet-2 is evaluated on "harder" data based on HRRR's performance.

We do an approximate comparison of MetNet-2 also with the MetNet model [28]. An exact comparison is difficult due to changes in the projection of the input data that has architectural implications, as well as other differences in the test data. For this reason, we simply plot MetNet-2' and MetNet' performances on the same plot, despite the fact that they are evaluated on slightly different test datasets. MetNet was shown to exceed HRRR's performance up to 7 to 8 hours of lead time. MetNet-2 without the assimilation variables exceeds HRRR's performance for up to nearly 12 hours of lead time, as can be seen in Supplementary Figure 12. This extension to 12 hours comes purely from the better architecture of MetNet-2 and an improved training regime. Using the assimilation variables as input, MetNet-2 provides a further improvement and its performance is even higher with respect to that of MetNet across the full range (Supplementary Figure 13). This is despite the fact that the baseline HRRR achieves higher F1 scores on MetNet's test data than on MetNet-2's test data and finds MetNet's test data easier to predict well.

## F.4 Forms of Lead Time Conditioning

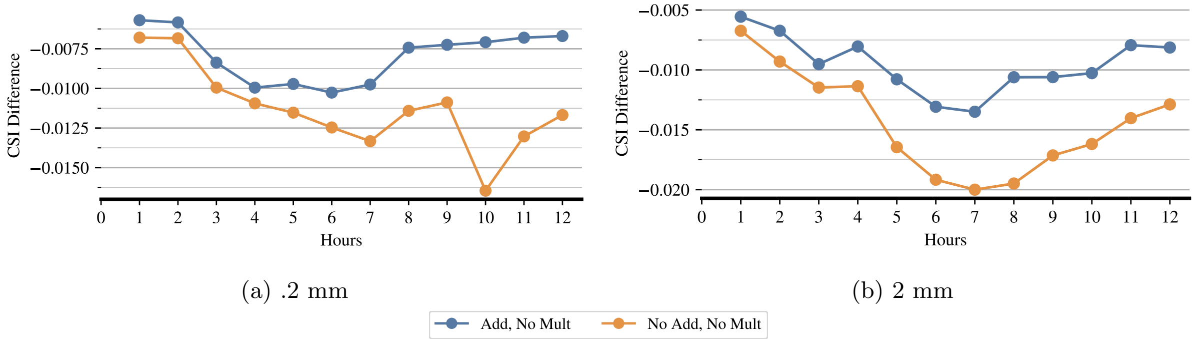


(a) .2 mm (b) 2 mm

Supplementary Figure 14: Ablation experiments with the adopted additive and multiplicative conditioning as baseline (Add, Mult) compared to only an additive variant (Add, No Mult) and the default concatenative variant (No Add, No Mult) used in Sønderby et al. [28]. Results on instantaneous precipitation.

The importance of the proposed, richer form of lead time conditioning also shows in the evaluation (Supplementary Figure 14). We compare it with the vanilla form of conditioning of simply concatenating the lead time index at the input [28] as well as part of the richer form of conditioning that only includes the additive bias component that shifts the activations in the convolutional layers. The proposed richer form of conditioning significantly improves performance with up to 1.5 CSI points over the default baseline and both the additive and the multiplicative components are important to the final performance.

## F.5 Dilation Factors

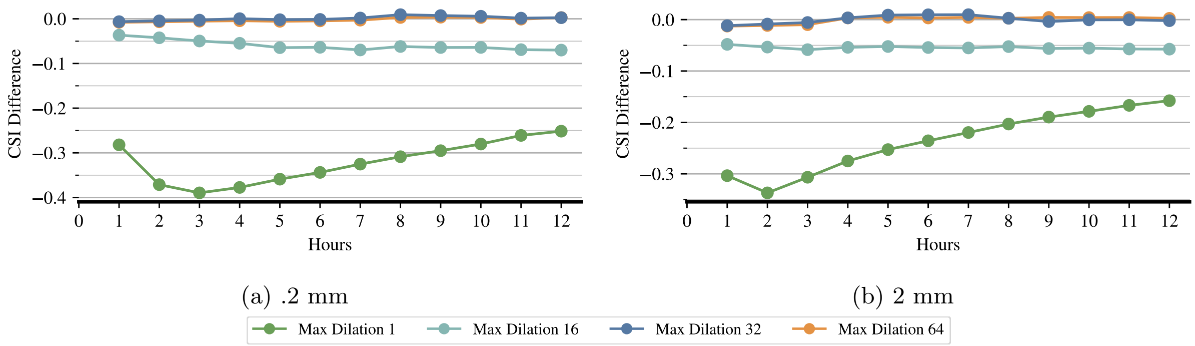


(a) .2 mm (b) 2 mm

Supplementary Figure 15: Ablation experiments for the size of the maximal dilation in the convolutions of MetNet-2's encoders (baseline dilation is 128). At maximal dilation of 16 one already sees a substantial drop in performance. Total depth of model and number of parameters is kept the same across all experiments.

One important feature of the MetNet-2 system and architecture is the necessity to process information from a large spatial context. On the one hand, the network's input must correspond to a large spatial context and, on the other, the network's connectivity must ensure that each point on the target patch has access to the portion of the input spatial context that it needs for a good forecast. The key aspect of the architecture that aims at capturing a large context for each of the target patch locations is the pattern of exponentially increasing factors of dilation. In this set of experiments, we ablate the maximal size of these factors, from 128 that is the default to 64, 32 and 16, as well as no dilation at all. Note that the difference between 64 and 128 is just one more residual block of larger dilation, which in the 64 version is replaced by additional layers with smaller dilation. Thus substantial differences in captured context size only appear when the maximum dilation is significantly smaller than 128. Supplementary Figure 15 summarizes the respective results for

the MetNet-2 model. We see that the performance of the architecture tends to decrease as the maximum dilation factors get smaller. This is especially marked at later lead times when processing a large spatial context effectively becomes necessary for a good forecast. These results indicate that the unusually large dilation factors rarely used in spatial convolutions succeed at capturing large context effectively and are an important factor in MetNet-2's model performance.

# G Supplement: Interpretation

The 100s of weather features used as input into MetNet-2 make it important to understand what a physics-free machine learning model, such as MetNet-2, is learning.

We aim to interpret MetNet-2 in order to explain individual predictions (local interpretability) as well as explaining phenomena learnt by the model globally, i.e. across all samples. Local interpretability helps us answer questions such as which spatial region of an input feature was crucial for forecasting the output. Whereas, global interpretability can help us identify useful minimal subsets of features and their physical interactions.

We obtain attributions using a technique called Integrated Gradients [29] that allows attributing the prediction $f(x)$ to the inputs $x$. The gradients are computed for each prediction at points along a straightline path from the chosen baseline to the actual input, and then aggregated. More formally,

$$IntegratedGrads(x) ::= (x - x') \times \int_{\alpha=0}^{1} \frac{\partial f(x_\alpha)}{\partial x} d\alpha \quad (5)$$

where $x_\alpha = x' + \alpha \times (x - x')$, is a point on the straightline path. Sundararajan et al. [29] suggest that the baseline chosen should be such that the prediction at that baseline should be neutral. Integrating the gradients from a neutral prediction to the actual prediction helps us understand which inputs were important for the actual prediction. In our case, we choose a baseline $x'$ such that the precipitation prediction, made by MetNet-2, at $x'$ is as close to zero probability of rain as possible, meaning a neutral prediction. We use the minimum value of each input feature as its baseline input to get this close to zero probability prediction.

Attribution maps have been typically used for singular classification outputs, and this is be one of the first applications of this technique to a model with a large multi-dimensional output. McGovern et al. [16] discuss various techniques for explaining black box ML models on various weather prediction tasks. However, all of the applications involve a single predicted output for each class. In our case, while we can obtain the attributions for each predicted pixel that represents a 1 km $\times$ 1 km, it will be humanly impossible to consume and make sense of the attribution maps for each pixel in the output separately. Additionally, the complexity of most of the existing interpretability tools increases greatly if they were to be applied to every single predicted pixel in a multi-dimensional output, separately. We, therefore, use Integrated Gradients to explain not just a single predicted pixel but the entire multi-dimensional output of predictions for the given input. For global interpretability, we aggregate per-feature attributions across space and over all the predictions and samples. This essentially gives us a single attribution value for each feature that represents the relative importance of that feature to the model.

In simpler terms, an attribution value for any given single input pixel represents how much it contributes to a single pixel probability prediction. The feature importance analysis computes how much on average, across all samples, each input contributes to a single predicted pixel. When doing a per-sample analysis, we compute how much on average, the input contributes to a predicted pixel for that specific sample. The attribution values for each of the features are, therefore, comparable to each other.

## G.1 Analysis of MRMS Radar and GOES Satellites

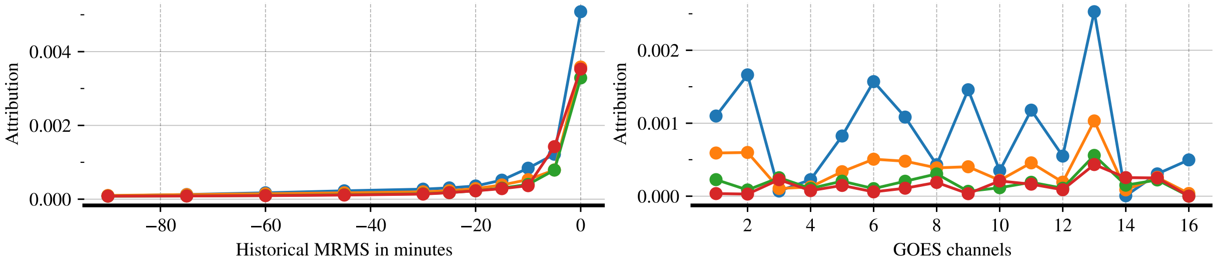


(a) MRMS (b) GOES

Supplementary Figure 16: Importance of MRMS and GOES at different forecast hours. The attribution values correspond to how much on average, across all samples, each input pixel contributes to a single predicted pixel.

Supplementary Figure 16 shows the attribution of MRMS and GOES historical data for different forecast hours. It is evident that both MRMS and GOES are highly important for nowcasting, i.e., 1-3 hr forecasts, and decrease in importance for the longer forecast hours. In alignment with our expectation, the model learns most from the latest (0th minute) MRMS data, and there is a sharp drop for all other historical data points.

In Supplementary Figure 16b, we see that ABI Band 13 in GOES, the *clean infrared window* 10.3 µm band, gets the highest attribution. According to NOAA [1], this band is least sensitive to water vapor absorption than other infrared bands. It therefore improves atmospheric moisture corrections, aids in cloud and other atmospheric feature identification/classification. This shows that the model has learnt from features that conforms with our knowledge of what the most important GOES band should be. It is interesting to note that ABI Band 2, the *Red Visible* 0.64 µm band, is important for the 1 hour forecast, but rapidly decreases in importance for even the 3 hour forecast, and has no significance to any of the longer hours. This could be due to the very fine resolution (0.5 km) of this band which allows detection of boundaries and small clouds making it useful for nowcasting. In contrast, ABI Band 3, the *Veggie* 0.86 textmugreek m band, has higher attribution for the longer range forecasts than for the 1 hr nowcast, implying that the land characteristics maybe an important feature predicting rain a few hours out. The *Mid-level water vapour* 6.9 textmugreek m band, ABI Band 9, is used for tracking middle tropospheric winds, monitoring severe weather potential, estimating mid-level moisture, and other purposes [26]. That the prediction of MetNet-2 relies on this feature is another sanity check that the model is learning meaningful information.

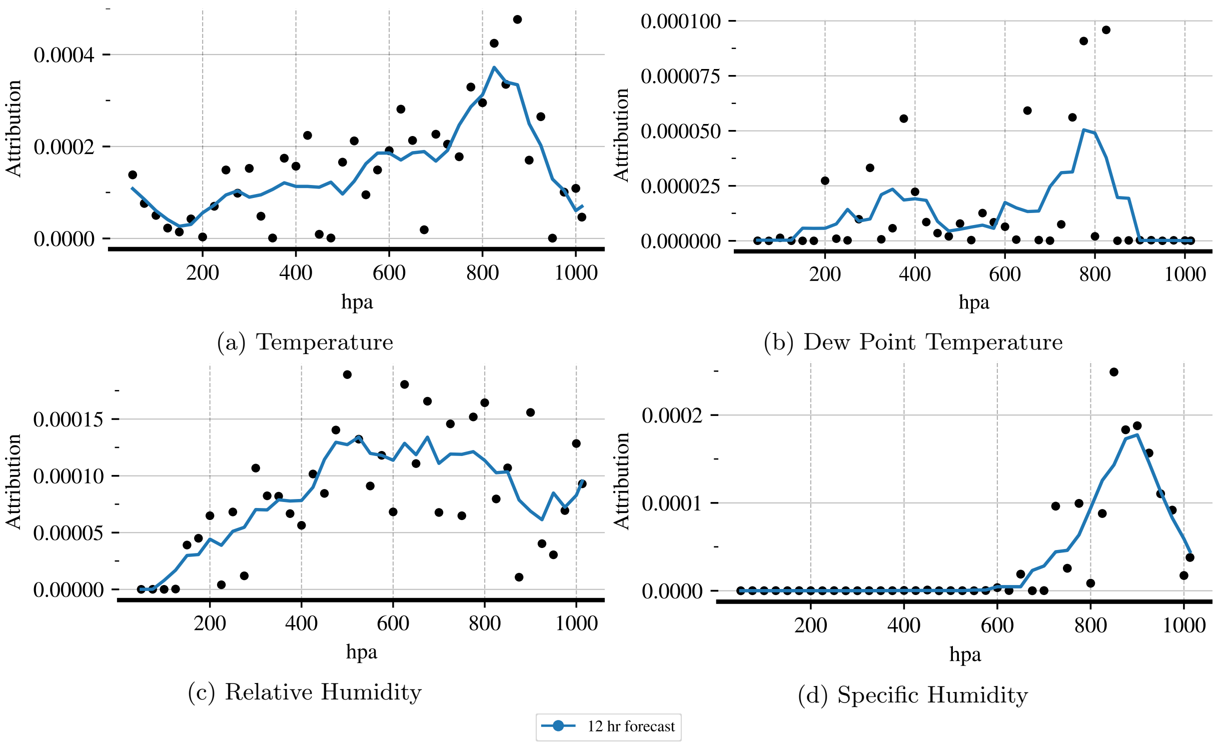


(a) Temperature (b) Dew Point Temperature (c) Relative Humidity (d) Specific Humidity

Supplementary Figure 17: Attribution of different weather features at 12 hour forecast.

## G.2 Analysis of Assimilation

Including the Assimilaton features as an input was significantly beneficial to MetNet-2 (Supplementary Figure 12). These features included the surface level weather features as well as the data at pressure levels ranging from 50 hPa to 1013 hPa. Supplementary Figure 17 plots the attribution of some of these features at 40 different pressure levels in this range, from just above the troposphere to sea level.

In a noteworthy observation, Figure 8a in the main text shows that absolute vorticity at pressure levels near 250 hPa is important for twelve hour precipitation forecasts. Absolute vorticity is the curl of the horizontal winds, so it can be considered a feature combining the effects of the u- and v-components of the wind. Figure 8b shows that the relative importance of absolute vorticity is small for near-term forecasts, but grows in importance as lead time increases. The importance of upper-level vorticity for a twelve hour forecast is consistent with quasi-geostrophic theory (QG theory). QG theory is a set of simplifications and filtering of the equations of motion, and a key result is that positive vorticity in the upper-troposphere is consistent with upward motion in the lower-troposphere [6]. This upward motion, does not directly trigger precipitation, but prepares the atmosphere for convection.

Supplementary Figure 17a is a plot of the attribution of temperature at different pressure levels, with a peak at ≈775 - 850 hPa. Temperature at 850 hPa, approximately 1.5 km above sea level and over the atmospheric boundary layer, generally used for detection of warm and cold fronts. At this height, the temperature sees no daily variations and effects of cooler surfaces such as the ocean are minimal.

The attribution plot of dew point temperature, Supplementary Figure 17b, shows two peaks at 375 hPa and 775 hPa, indicating the moisture in the upper level and lower level of the atmosphere is predictive of precipitation 12 hours out.

In Supplementary Figure 17c and Supplementary Figure 17d, we can see a comparison of attributions between relative humidity and specific humidity, respectively. Specific humidity levels in the lower levels of the atmosphere indicate how much moisture there is for formation of storms, higher the moisture, higher the precipitation rates will be. Relative humidity is likely indicating large-scale cloudiness.

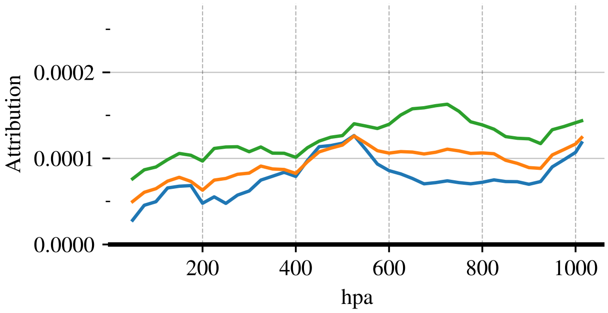


(a) U-component of wind

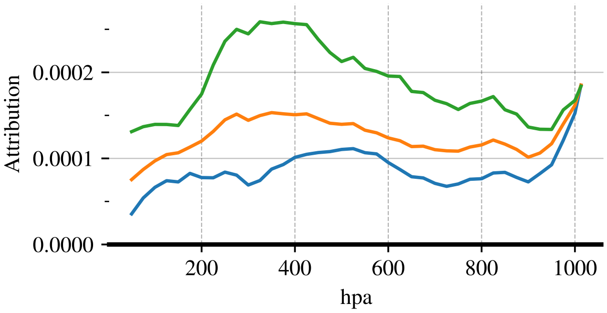


(b) V-component of wind




Supplementary Figure 18: Attribution of wind over different forecast hours and pressure.

In Supplementary Figure 18, we look at how the attributions change for the u and v-component of wind.

For a 1hr forecast, surface level wind is most important for both components. But beyond the 4hr forecast, notice that v-component of the surface wind has substantially lesser importance than that in the upper troposphere (200-400 hPa). The v-component, the meridional flow of winds, has higher importance in the upper troposphere. Significant values of this component in the upper level indicates a low that is favorable for producing precipitation. Meridional flow aloft can be found near upper-level lows, which are favorable for large scale precipitation.

It is expected that the u-component, flow of horizontal wind towards East, is not as important in the upper troposphere since it represents how fast the wind blowing.

We also noticed that for 1 hour nowcasting, features such as snow mixing ratio, cloud water mixing ratio had greater relative importance than at later hours. From this the model essentially learns that if it is raining now then it will continue to rain in the near future. MetNet-2 has learned the technique of persistence.

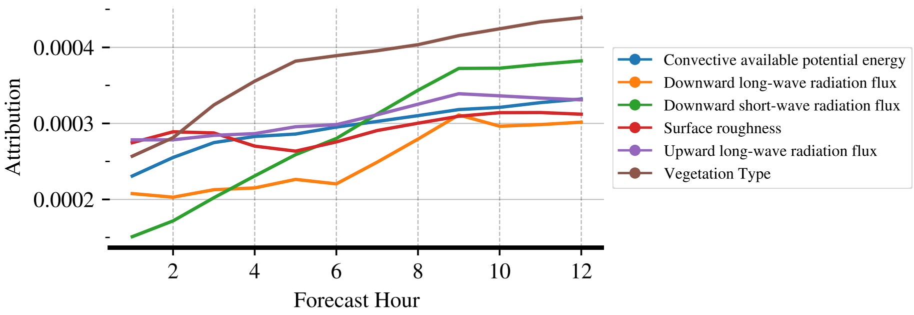


(a)

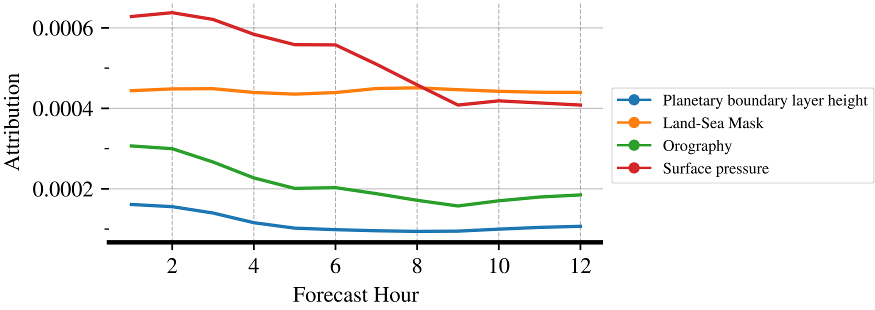


(b)

Supplementary Figure 19: Surface features increasing (a) or decreasing (b) in importance with increasing forecast hour.

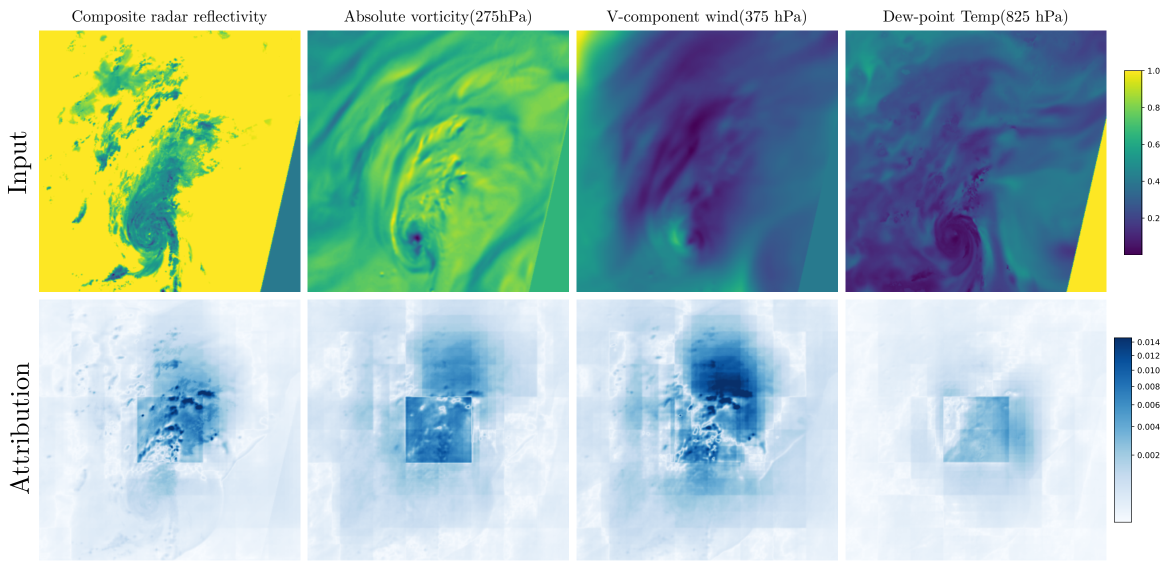


Supplementary Figure 20: Attribution maps for MetNet-2's prediction of Hurricane Isaias shown in Figure 4b. Each attribution pixel represents the contribution of the corresponding input pixel, on average across 128X128 pixels of the target.

We also looked at behaviors of various surface level features. Supplementary Figure 19a plots features that increased in importance with increasing length of forecast and, in contrast, Supplementary Figure 19b plots features that decreased in importance. Downward short-wave radiation flux together with the upward long wave radiation flux indicate how much sunlight is reaching the surface, thereby warming it. When there is not enough sunlight and surface temperatures remain cool, deep convective storms are unlikely. Downward long-wave radiation, on the other hand, is the heat emitted back to Earth from the atmosphere, indicating how much cloud cover is currently present, which is useful for nowcasting. Planetary boundary layer height varies throughout the day and is therefore less reliable for what can happen a 12 hours later. Similarly, the surface pressure indicates where the storms are forming in the present, being more useful for nowcasting.

### G.3 Local Interpretability Results

To analyze the pixels in each sample that MetNet-2 was keying in on, we plotted attribution maps for every input feature. Supplementary Figure 20, shows attributions of the 12 hour forecast for features–radar reflectivity, absolute vorticity at 275 hPa and the v-component of wind at 375 hPa. For all of these, the entire context was influential, with pixels closer to the center of the sample being more important than those at the borders. The weather features that were unimportant to the prediction has attribution maps with values close to zero.

## H Supplement: Regional Evaluation

We evaluate all our models also on a diverse set of sub-regions of CONUS using the same radar targets that we use on the full CONUS evaluation. We choose this set of regions because they represent different climatological regimes of precipitation within CONUS while also broadly covering different geographical parts of CONUS.

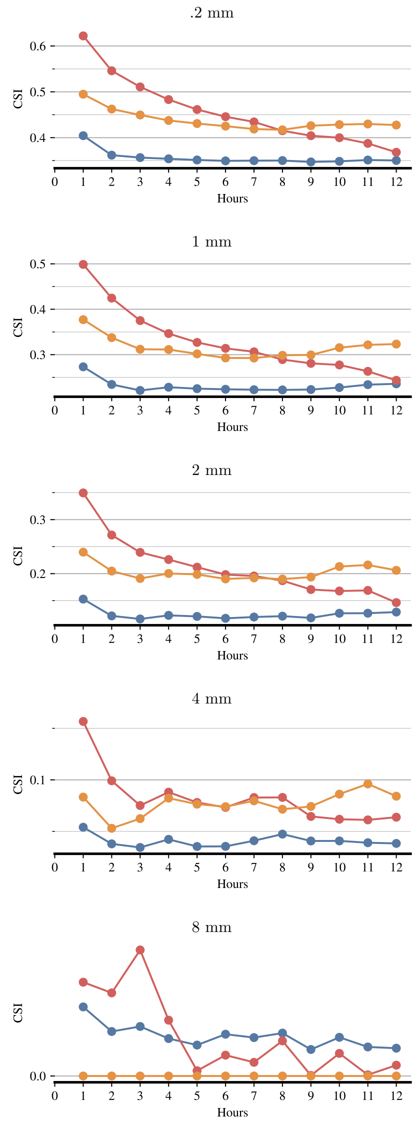


Supplementary Figure 21 (a): Pacific Northwest

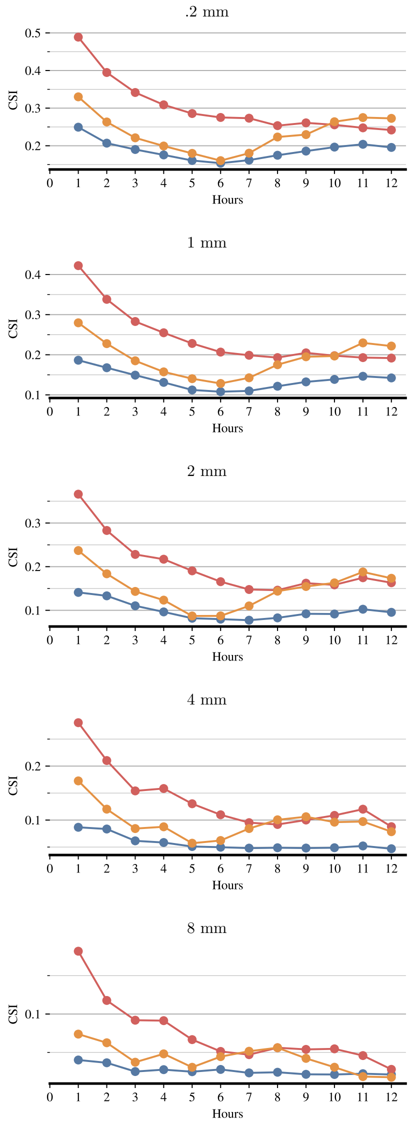


Supplementary Figure 21 (b): Florida

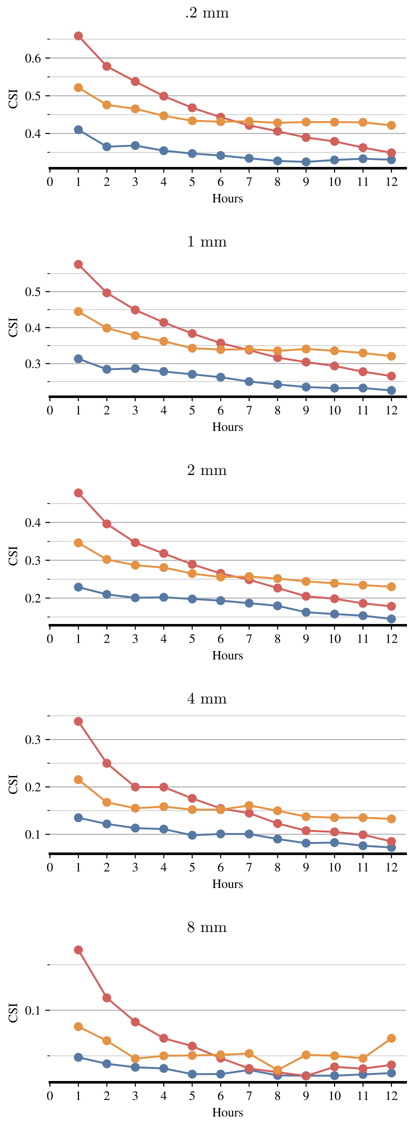


Supplementary Figure 21 (c): East Coast

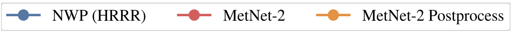


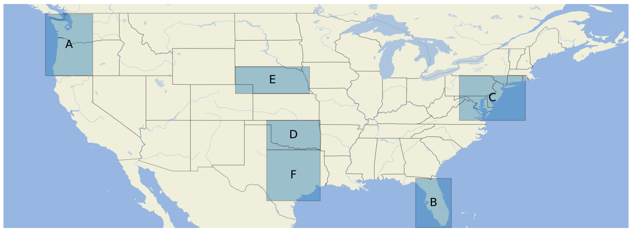

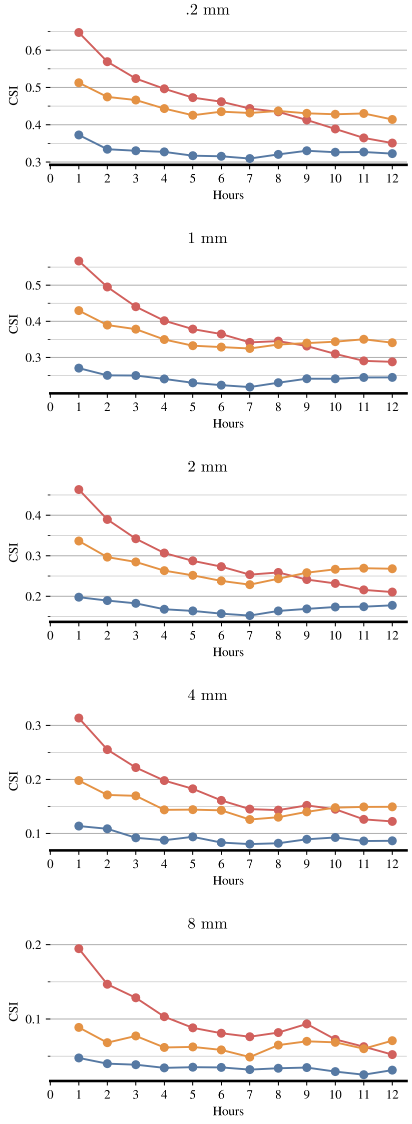


Supplementary Figure 21 (d): South Plains (Oklahoma)

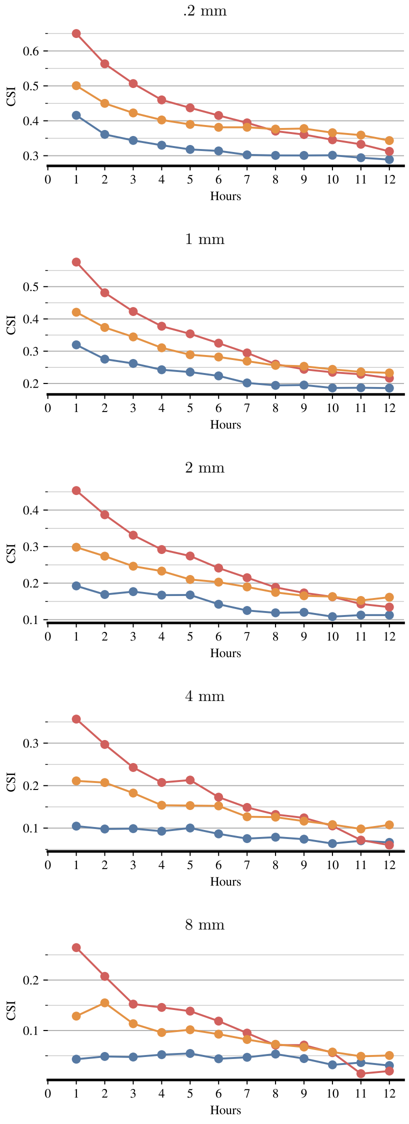


Supplementary Figure 21 (e): North Plains (Nebraska)

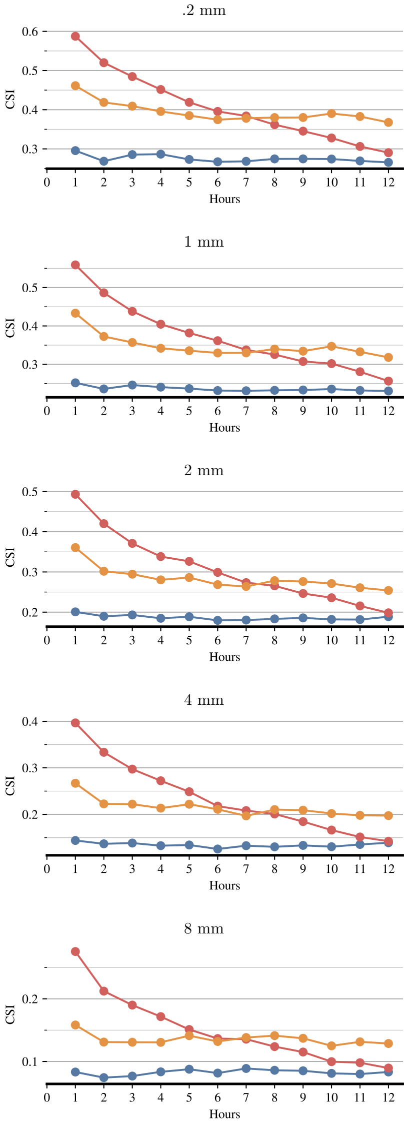


Supplementary Figure 21 (f): Texas

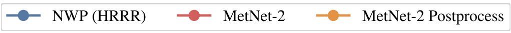

# References


[1] GOES ABI. Abi bands quick information guides. https://www.goes-r.gov/mission/ABI-bands-quick-info.html, 2021. Accessed: 2021-06-01.

[2] Shreya Agrawal, Luke Barrington, Carla Bromberg, John Burge, Cenk Gazen, and Jason Hickey. Machine learning for precipitation nowcasting from radar images. *arXiv preprint arXiv:1912.12132*, 2019.

[3] Antoine Alléon, Grégoire Jauvion, Boris Quennehen, and David Lissmyr. Plumenet: Large-scale air quality forecasting using a convolutional lstm network, 2020.

[4] Peter Bauer, Alan Thorpe, and Gilbert Brunet. The quiet revolution of numerical weather prediction. *Nature*, 525(7567):47–55, 2015.

[5] Stanley G. Benjamin, Stephen S. Weygandt, John M. Brown, Ming Hu, Curtis R. Alexander, Tatiana G. Smirnova, Joseph B. Olson, Eric P. James, David C. Dowell, Georg A. Grell, Haidao Lin, Steven E. Peckham, Tracy Lorraine Smith, William R. Moninger, Jaymes S. Kenyon, and Geoffrey S. Manikin. A north american hourly assimilation and model forecast cycle: The rapid refresh. *Monthly Weather Review*, 144(4):1669 – 1694, 2016.

[6] Howard B Bluestein. *Synoptic-Dynamic Meteorology in Midlatitudes: Observations and Theory of Weather Systems*, volume 1. Oxford University Press, New York, NY USA, 1992.

[7] James Bradbury, Roy Frostig, Peter Hawkins, Matthew James Johnson, Chris Leary, Dougal Maclaurin, George Necula, Adam Paszke, Jake VanderPlas, Skye Wanderman-Milne, and Qiao Zhang. JAX: composable transformations of Python+NumPy programs, 2018.

[8] Glenn W. Brier. Verification of forecasts expressed in terms of probability. *Monthly Weather Review*, 78(1):1–3, 1950.

[9] Matthew Chantry, Hannah Christensen, Peter Dueben, and Tim Palmer. Opportunities and challenges for machine learning in weather and climate modelling: hard, medium and soft ai. *Philosophical Transactions of the Royal Society A: Mathematical, Physical and Engineering Sciences*, 2021.

[10] GOES. Noaa geostationary satellite (goes). https://www.goes.noaa.gov/index.html, 2021. Accessed: 2021-06-01.

[11] Y. Ham, Jeong hwan Kim, and Jing-Jia Luo. Deep learning for multi-year enso forecasts. *Nature*, pages 1–5, 2019.

[12] Hans Hersbach. Decomposition of the continuous ranked probability score for ensemble prediction systems. *Weather and Forecasting*, 15(5):559 – 570, 2000.

[13] Sepp Hochreiter and Jürgen Schmidhuber. Long short-term memory. *Neural computation*, 9:1735–80, 12 1997.

[14] HRRR. Hrrr state variables. https://home.chpc.utah.edu/~u0553130/Brian_Blaylock/HRRR_archive/hrrr_prs_table_f00-f01.html, 2021. Accessed: 2021-12-01.

[15] Nal Kalchbrenner, Lasse Espeholt, Karen Simonyan, Aaron van den Oord, Alex Graves, and Koray Kavukcuoglu. Neural machine translation in linear time, 2017.

[16] Amy McGovern, Ryan Lagerquist, David John Gagne, G. Eli Jergensen, Kimberly L. Elmore, Cameron R. Homeyer, and Travis Smith. Making the black box more transparent: Understanding the physical implications of machine learning. *Bulletin of the American Meteorological Society*, 100(11):2175 – 2199, 2019.

[17] MRMS. Multi-radar/multi-sensor system (mrms). https://www.nssl.noaa.gov/projects/mrms/, 2021. Accessed: 2021-06-01.

[18] Ethan Perez, Florian Strub, Harm De Vries, Vincent Dumoulin, and Aaron Courville. Film: Visual reasoning with a general conditioning layer. In *Proceedings of the AAAI Conference on Artificial Intelligence*, volume 32, 2018.

[19] Rachel Prudden, Samantha Adams, Dmitry Kangin, Niall Robinson, Suman Ravuri, Shakir Mohamed, and Alberto Arribas. A review of radar-based nowcasting of precipitation and applicable machine learning techniques, 2020.

[20] Stephan Rasp and Sebastian Lerch. Neural networks for postprocessing ensemble weather forecasts. *Monthly Weather Review*, 146(11):3885 – 3900, 2018.

[21] Suman V. Ravuri, Karel Lenc, Matthew Willson, Dmitry Kangin, Rémi Lam, Piotr Mirowski, Megan Fitzsimons, Maria Athanassiadou, Sheleem Kashem, Sam Madge, Rachel Prudden, Amol Mandhane, Aidan Clark, Andrew Brock, Karen Simonyan, Raia Hadsell, Niall H. Robinson, Ellen Clancy, Alberto Arribas, and Shakir Mohamed. Skillful precipitation nowcasting using deep generative models of radar. *CoRR*, abs/2104.00954, 2021.

[22] R.J. Donaldson, R.M. Dyer, and M.J. Kraus. An objective evaluator of techniques for predicting severe weather events. In *Preprints, Ninth Conference on Severe Local Storms*, Norman, OK USA, 1975. American Meteorological Society.

[23] Lans P. Rothfusz, Russell Schneider, David Novak, Kimberly Klockow-McClain, Alan E. Gerard, Chris Karstens, Gregory J. Stumpf, and Travis M. Smith. Facets: A proposed next-generation paradigm for high-impact weather forecasting. *Bulletin of the American Meteorological Society*, 99(10):2025 – 2043, 2018.

[24] Lans P. Rothfusz, Russell Schneider, David Novak, Kimberly Klockow-McClain, Alan E. Gerard, Chris Karstens, Gregory J. Stumpf, and Travis M. Smith. FACETs: A Proposed Next-Generation Paradigm for High-Impact Weather Forecasting. *Bulletin of the American Meteorological Society*, 99(10):2025–2043, October 2018.

[25] Tom Schaul, John Quan, Ioannis Antonoglou, and David Silver. Prioritized experience replay, 2016.

[26] Timothy J. Schmit, Paul Griffith, Mathew M. Gunshor, Jaime M. Daniels, Steven J. Goodman, and William J. Lebair. A Closer Look at the ABI on the GOES-R Series. *Bulletin of the American Meteorological Society*, 98(4):681 – 698, 2017.

[27] M. G. Schultz, C. Betancourt, B. Gong, F. Kleinert, M. Langguth, L. H. Leufen, A. Mozaffari, and S. Stadtler. Can deep learning beat numerical weather prediction? *Philosophical Transactions of the Royal Society A: Mathematical, Physical and Engineering Sciences*, 379(2194):20200097, 2021.

[28] Casper Kaae Sønderby, Lasse Espeholt, Jonathan Heek, Mostafa Dehghani, Avital Oliver, Tim Salimans, Shreya Agrawal, Jason Hickey, and Nal Kalchbrenner. Metnet: A neural weather model for precipitation forecasting. *arXiv preprint arXiv:2003.12140*, 2020.

[29] Mukund Sundararajan, Ankur Taly, and Qiqi Yan. Axiomatic attribution for deep networks. In *International Conference on Machine Learning*, pages 3319–3328. PMLR, 2017.

[30] Kevin Trebing and Siamak Mehrkanoon. Wind speed prediction using multidimensional convolutional neural networks. In *2020 IEEE Symposium Series on Computational Intelligence (SSCI)*, pages 713–720, 2020.

[31] Kevin Trebing, Tomasz Stanczyk, and Siamak Mehrkanoon. Smaat-unet: Precipitation nowcasting using a small attention-unet architecture. *Pattern Recognition Letters*, 145:178–186, 2021.

[32] Aaron van den Oord, Sander Dieleman, Heiga Zen, Karen Simonyan, Oriol Vinyals, Alex Graves, Nal Kalchbrenner, Andrew Senior, and Koray Kavukcuoglu. Wavenet: A generative model for raw audio, 2016.

[33] Shi Xingjian, Zhourong Chen, Hao Wang, Dit-Yan Yeung, Wai-Kin Wong, and Wang-chun Woo. Convolutional lstm network: A machine learning approach for precipitation nowcasting. In *Advances in neural information processing systems*, pages 802–810, 2015.

[34] Yuanzhong Xu, HyoukJoong Lee, Dehao Chen, Blake A. Hechtman, Yanping Huang, Rahul Joshi, Maxim Krikun, Dmitry Lepikhin, Andy Ly, Marcello Maggioni, Ruoming Pang, Noam Shazeer, Shibo Wang, Tao Wang, Yonghui Wu, and Zhifeng Chen. GSPMD: general and scalable parallelization for ML computation graphs. *CoRR*, abs/2105.04663, 2021.

[35] Jining Yan, Lin Mu, Lizhe Wang, R. Ranjan, and Albert Y. Zomaya. Temporal convolutional networks for the advance prediction of enso. *Scientific Reports*, 10, 2020.

[36] Jun-Ichi Yano, Micha Z. Ziemiaski, Mike Cullen, Piet Termonia, Jeanette Onvlee, Lisa Bengtsson, Alberto Carrassi, Richard Davy, Anna Deluca, Suzanne L. Gray, V?ctor Homar, Martin Kohler, Simon Krichak, Silas Michaelides, Vaughan T. J. Phillips, Pedro M. M. Soares, and Andrzej A. Wyszogrodzki. Scientific challenges of convective-scale numerical weather prediction. *Bulletin of the American Meteorological Society*, 99(4):699 – 710, 2018.
